# Continuous infusion of amphotericin B: preliminary experience at Faculdade de Medicina da Fundação ABC

**DOI:** 10.1590/S1516-31802005000500004

**Published:** 2005-09-01

**Authors:** Roberto Palermo Uehara, Victor Hugo Lara de Sá, Érika Tae Koshimura, Fernanda Vilas Boas Prudente, Luciana Tomanik Cardozo de Mello Tucunduva, Marina Sahade Gonçalves, Eliana Sueco Tibana Samano, Auro del Giglio

**Keywords:** Amphotericin B, Neutropenia, Fever, Hematology, Leukemia, Anfotericina B, Neutropenia, Febre, Hematologia, Leucemia

## Abstract

**CONTEXT AND OBJECTIVE:**

Intravenous amphotericin B deoxycholate (AmB-D) infusions, usually given over 4 hours, frequently induce nephrotoxicity and undesirable infusion-related side effects such as rigors and chills. There is evidence in the literature that the use of AmB-D in the form of continuous 24-hour infusion is less toxic than the usual four-hour infusion of this drug. Our objective was to evaluate the efficacy and safety of continuous infusion of AmB-D for the treatment of persistent fever in neutropenic patients with hematological malignancies after chemotherapy.

**DESIGN AND SETTING:**

Observational retrospective analysis of our experience with continuous infusion of AmB-D, at Faculdade de Medicina da Fundação ABC and Hospital Estadual Mário Covas in Santo André.

**METHODS:**

From October 2003 to May 2004, 12 patients with hematological malignancies and chemotherapy-induced neutropenia received 13 cycles of continuous infusion of AmB-D.

**RESULTS:**

The median dose of AmB-D was 0.84 mg/kg/day (0.33 to 2.30 mg/kg/day). Concomitant use of nephrotoxic medications occurred in 92% of the cycles. Nephrotoxicity occurred in 30.76% of the cycles, hypokalemia in 16.67%, hepatotoxicity in 30% and adverse infusion-related events in 23%. All patients survived for at least seven days after starting continuous infusion of AmB-D, and clinical resolution occurred in 76% of the cycles.

**CONCLUSIONS:**

Continuous infusion of AmB-D can be used in our Institution as an alternative to the more toxic four-hour infusion of AmB-D and possibly also as an alternative to the more expensive liposomal formulations of the drug.

## RESUMO

**CONTEXTO E OBJETIVO:**

Pacientes neutropênicos com febre persistente podem apresentar infecções fúngicas com freqüência. A administração de anfotericina B deoxicolato tem sido padrão para estes pacientes, no entanto sua infusão endovenosa, usualmente administrada em quatro horas, pode levar a nefrotoxicidade, hepatotoxicidade e efeitos adversos relacionados à infusão, como tremores e calafrios. A literatura evidencia que o uso de anfotericina B deoxicolato em infusão contínua de 24 horas pode ser menos tóxica em relação à administração usual. O objetivo do estudo foi avaliar a eficácia, segurança e toxicidade da anfotericina B infusional contínua em pacientes onco-hematológicos após quimioterapia com neutropenia febril persistente.

**TIPO DE ESTUDO E LOCAL:**

Estudo observacional e retrospectivo de nossa experiência com anfotericina B deoxicolato em infusão contínua de 24 horas, na Faculdade de Medicina da Fundação ABC e Hospital Estadual Mário Covas, em Santo André.

**MÉTODOS:**

No período entre outubro de 2003 e maio de 2004, 12 pacientes com neoplasias hematológicas e neutropenia febril induzida por quimioterapia receberam 13 ciclos de anfotericina B deoxicolato infusional.

**RESULTADOS:**

A dose média da infusão foi de 0,84 mg/kg/dia. O uso concomitante de outras drogas nefrotóxicas ocorreu em 92% dos ciclos. Foram observados nefrotoxicidade em 30,76%, hipocalemia em 16,67%, hepatotoxicidade em 30% e efeitos adversos relacionados à infusão em 23% dos ciclos. Todos os pacientes sobreviveram aos sete primeiros dias após o início do tratamento e a resolução clínica ocorreu em 76% dos ciclos.

**CONCLUSÃO:**

A infusão contínua de anfotericina B é exeqüível para uso em nossa instituição como alternativa à infusão em quatro horas (mais tóxica) e possivelmente às caras formulações lípidicas desta droga.

## INTRODUCTION

Invasive fungal infections have become a challenging issue with regard to neutropenic patients. Because of delays in diagnosing such infections, they are frequently fatal. Sometimes they are only diagnosed at the autopsy.^1^ Prolonged neutropenia is a major risk factor for invasive fungal infection. The incidence of invasive fungal infections ranges from 2% to 47%, depending on many other concomitant risk factors (e.g. the use of steroids or lymphoid malignancies).^2^

In the early 1980s, many trials drew attention to the empirical and prophylactic application of antifungal drugs within such a scenario. In fact, amphotericin B deoxycholate (AmB-D) in the setting of persistent fever in neutropenic patients reduces the relative risk for documented invasive fungal infections by 50% to 80% and the overall mortality rate by 23% to 45%.^2^ The use of AmB-D in these patients is, however, limited by the frequent induction of nephrotoxicity and by the undesirable infusion-related side effects such as fever, rigors and chills. Because of this adverse toxicity profile, AmB-D has been compared unfavorably with new amphotericin formulations and other antifungal drugs.^2^

In the late 1990s, liposomal amphotericin B (L-AmB) emerged as a new option for the treatment for persistent fever in neutropenic patients by reducing the frequency of proven breakthrough fungal infections, with lower infusion-related toxic reactions and also reduced nephrotoxicity,^2–4^ in comparison with AmB-D. Recently, other antifungal drugs, such as new triazoles and caspofungin, have emerged as promising agents for the prevention of invasive fungal infections in persistently febrile neutropenic patients.^2–4^ In fact, in a comparative trial, voriconazole, a second-generation triazole, proved to be a suitable alternative to amphotericin B preparations.^4^ The major inconvenience of these new antifungal drugs, however, is their higher cost, which may seriously hamper their use in underdeveloped countries.

Recently, a small randomized trial comparing continuous intravenous versus four-hour infusion of AmB-D showed a lower incidence of infusion related events and nephrotoxicity among the patients who received continuous intravenous infusion.^5^ Following the publication of these results, we also started to use AmB-D in the form of continuous 24-hour infusion. Here, we report on our preliminary experience with continuous infusion of AmB-D at our center, in terms of its efficacy and toxicity.

## OBJECTIVE

To evaluate the efficacy, safety and toxicity of continuous infusion of AmB-D in febrile patients with neutropenia consequent to undergoing chemotherapy for hematological malignancies.

## PATIENTS AND METHODS

This observational study was conducted in Faculdade de Medicina da Fundação ABC and Hospital Estadual Mário Covas in Santo André, which is a public, teaching healthcare institution, between October 2003 and May 2004.

Two of the initial 15 patients were excluded from the analyses. Two of these had another antifungal agent administered together with the continuous infusion of AmB-D, and the other had not received previous systemic chemotherapy. One patient received two cycles of continuous infusion of AmB-D and was analyzed twice, as each admission was considered as a separate event. A total of 13 procedures of continuous infusion of AmB-D were therefore administered.

The records of all 12 patients were retrospectively reviewed. The presence of toxicity and absence of breakthrough infection was verified according to the following criteria proposed by Walsh:^4^

- Age between 15 and 70 years;
- Indication for receiving intravenous amphotericin B;
- Myelosuppressive systemic chemotherapy was previously administered;
- Persistent febrile neutropenia (absolute neutrophil count (ANC) of less than 1000/mm^3^ and temperature ≥ 37.8° C);
- At least five days of prior use of empirical broad-spectrum antibiotics;
- Normal renal function before intravenous AmB was started;
- Alanine aminotransferase and aspartate aminotransferase levels of no more than three times baseline;
- Bilirubin level of no more than three times baseline;
- No other antifungal agents, except for previous prophylaxis (which had to be discontinued prior to starting the continuous infusion of AmB-D).

Three of the initial 15 patients were excluded from the analyses. Two of these had another antifungal agent administered together with the continuous infusion of AmB-D, and the other had not received previous systemic chemotherapy. One patient received two cycles of continuous infusion of AmB-D and was analyzed twice as each admission was considered as a separate event. A total of 13 procedures of continuous infusion of AmB-D were therefore administered.

The records of all 12 patients were retrospectively reviewed. The presence of toxicity and absence of breakthrough infection was verified according to the following criteria proposed by Walsh:^4^

- Creatinine level increased to 1.5 times baseline value;
- Hypokalemia ≤ 2.5 mmol/l;
- Hypomagnesemia ≤ 0.6 mmol/l;
- Increased bilirubin levels;
- Aspartate aminotransferase (AST) or alanine aminotransferase (ALT) level of more than five times baseline values, if these values were greater than the reference range. Hepatotoxicity was also deemed to be present when AST or ALT was three times more than baseline, if the baseline was between two and five times the normal range. On the other hand, if the baseline was between 5 and 10 times the normal range, AST or ALT only had to reach twice the baseline value for hepatotoxicity to be deemed present. The baseline values were considered to be the laboratory values obtained immediately before the continuous infusion of AmB-D was started.

Because of drug stability concerns, the total dose of AmB-D required (mg/kg/day) was divided into four doses, and each of these was given as a 6-hour infusion procedure. Each quarter of the total dose was thus given in the form of 250 ml of 5% glucose solution, without any additives.

The infusion-related toxic reactions were retrospectively estimated from the frequency with which our Institution's protocol for the treatment of fever, chills and rigors secondary to AmB-D infusion was utilized (i.e. considering any use of acetaminophen, diphenhydramine, steroids or meperidine during the period of continuous infusion of AmB-D). Concomitant use of other nephrotoxic drugs was defined as the simultaneous use of amino-glycoside, foscarnet or cyclosporin.

The degree of certainty of the diagnosis of fungal infection was defined according to the Walsh criteria:^4^

- Proven: by biopsy or bronchoalveolar lavage;
- Probable: by radiologically typical lesions;
- Fungemia: positive blood culture;
- Possible: none of the above.

The primary efficacy of the treatment was defined as a composite of the following criteria, as proposed by Walsh:^4^

- Survival for 7 days after starting to use the study drug;
- Resolution of fever during the period of neutropenia;
- Successful treatment of any baseline fungal infection, if present;
- Absence of breakthrough fungal infection during administration of the study drug or within seven days after the completion of treatment;
- Absence of premature discontinuation of the drug because of toxicity or lack of efficacy.

## RESULTS

Among the patients included, 76.9% were female, the mean age was 36.23 years and 61.5% had acute myelogenous leukemia as the diagnosis (Table 1).

**Table 1.** Demographic characteristics of patients undergoing chemotherapy for hematological malignancies

Characteristics of the patients evaluated	Results
**Mean age** (n = 13)	36.23 years
**Sex** (n = 13)	
• Male	23.1%
• Female	76.9%
**Race** (n = 8)	
• White	37.5%
• Black	50%
• Other	12.5%
**Diagnosis** (n = 13)	
• Acute myelogenous leukemia	61.5%
• Non-Hodgkin's lymphoma (NHL)	23.1%
• Acute lymphocytic leukemia/NHL	15.4%
**Site of infection** (n = 13)	
• Indeterminate	61.5%
• Tonsils	7.7%
• Skin	7.7%
• Lung	7.7%
• Blood stream	15.4%
**Fungal diagnosis criteria**^4^ (n = 13)	
• Positive by bronchoalveolar lavage	15.4%
• Suggestive by radiologically typical lesions findings	53.8%
• Fungemia by positive blood culture	7.7%
• Possible	23.1%

In 61.5% of the cases, the site of fungal infection could not be determined. In 53.8%, the presumptive diagnosis was based on radiographic findings, while bronchoalveolar lavage/sputum was positive in 15.4%, and positive blood culture occurred in only 7.7% of the cases (Table 1).

The mean length of time since the previous broad-spectrum antibacterial therapy was 18.23 days, and a mean of 3.84 antibiotics had previously been administered to each patient. Prophylactic fluconazole had been prescribed for 61.5% of the patients. The mean rate for the continuous infusion of AmB-D was 0.84 mg/kg/day (range from 0.33 to 2.30 mg/kg/day).

In 30.76% of the patients, the creatinine level increased to 1.5 times baseline, but 92.4% of them were also taking other nephrotoxic drugs (aminoglycoside in all cases). Adverse infusion-related events were present in 23% of the cycles and hepatotoxicity in 30% of them (Table 2).

**Table 2.** Comparison of efficacy, safety and infusion-related toxic effect of amphotericin B in patients with hematological malignancies

	Continuous infusion of amphotericin B deoxycholate (present study)	Continuous infusion of amphotericin B deoxycholate (Eriksson et al.^5^)	Amphotericin B deoxycholate (Walsh et al.^3^)	Liposomal amphotericin B (Walsh et al.^3^)
**Creatinine > 1.5 x baseline**	30.76%	33%	49.40%	29.40%
**Other concomitant nephrotoxic drugs**	92.40%	28%	15.20%	6.30%
**Hypokalemia (≤ 2.5)**	16.67%	10%	11.60%	6.70%
**Hepatotoxicity**	30%	-	20.30%	17.80%
**Infusion-related effects (chills)**	23.07%	13%	73.5%	37.6%

With regard to the efficacy criteria, 100% of the patients were alive one week after continuous infusion of AmB-D started. Three fungal infections were documented, and two of these (66.60%) resolved completely. Breakthrough fungal infection was absent in 92.30% of the cases. There was no discontinuation of the drug due to toxicity or absence of efficacy, nor was there any need for association with another antifungal agent. In 76.92% of the cases, the fever resolved while the patient was still in the neutropenic period (Table 3).

**Table 3.** Comparative primary efficacy endpoints of amphotericin formulations in neutropenic patients

Efficacy end point	Continuous infusion of amphotericin B deoxycholate	Amphotericin B deoxycholate (Walsh et al.^3^)	Liposomal amphotericin B (Walsh et al.^3^)
**1- Seven-day survival following infusion of amphotericin B deoxycholate**	100%	89.50%	92.70%
**2- Successful treatment of baseline fungal infection**	66.60%	72.70%	81.80%
**3- Absence of breakthrough infection during the study period**	92.30%	89.20%	90.10%
**4- Absence of premature discontinuation due to loss of efficacy or high toxicity**	0%	18.60%	14.3%
**5- Resolution of fever during neutropenia**	76.92%	58.10%	58%

## DISCUSSION

Fungal infections within a scenario of neutropenia are always a challenge in caring for cancer patients, especially after chemotherapy. Because of the potential for high morbidity and mortality, any delay in diagnosing such infections may be fatal. Invasive fungal infection rates have ranged from 3% to 42% in patients with acute myelogenous leukemia treated with cytarabine and anthracycline-based chemotherapeutic regimens, and the mortality rates may reach 6% to 60%.^6^

During the last twenty years many different drugs have been developed to ameliorate and control fungal infections. On the other hand, economic issues related to this new formulations have been voiced. In the 1990s, L-AmB emerged as an alternative for the treatment of febrile neutropenic patients and was shown to be as effective as AmB-D and to cause lower infusion-related toxicity, less nephrotoxicity and fewer breakthrough fungal infections.^3^ Recently, a randomized trial was reported in which, despite the high cost of L-AmB, the pharmacoeconomic issues favored the use of L-AmB for patients at a high risk of developing renal failure. It was also suggested that to change patients from AmB-D to L-AmB after high levels of toxicity have developed may not be the most cost-effective strategy.^7^

Amphotericin B formulations have been compared in a systematic review, in which it was shown that L-AmB significantly reduced the risk of all causes of mortality by an estimated 28%, compared with conventional AmB-D (odds ratio, OR = 0.72; confidence interval, CI: 0.54 to 0.97). There was no significant difference in efficacy between L-AmB and AmB-D (OR = 1.21; CI: 0.98 to 1.49). Amphotericin B lipid complex and L-AmB were found to significantly reduce the risk of doubling the serum creatinine level, by an estimated 58% (OR = 0.42; CI 0.33 to 0.54). Comparing the lipid-based formulations with conventional AmB-D, the overall number of patients needed to treat (NNT) in order to prevent one death was 31. The NNT in order to prevent the doubling of the serum creatinine level for both amphotericin B lipid complex and L-AmB, in comparison with AmB-D, was six patients.^8^

Although some studies have supported the use of L-AmB as a standard antifungal agent, on the basis of its efficacy and from cost effectiveness analyses, many institutions have to limit their routine use of this practice because of the very high cost of L-AmB. Interestingly, in a recent randomized controlled trial that compared the use of 4 or 24-hour infusion of AmB-D, the 24-hour infusion of AmB-D reduced nephrotoxicity and the side effects related to infusion, without increased mortality.^5^ Continuous infusion of AmB-D therefore emerges as an interesting investigational alternative in the recent literature because of its promisingly low toxicity profile, effective antifungal activity and significantly lower cost. In fact, one milligram of L-AmB is about 35 times more expensive than one milligram of AmB-D (data not presented).

In our observational study, continuous infusion of AmB-D produced nephrotoxicity in 30.75% of the cycles, which was similar to the rate reported by Eriksson^5^ (33%) with 24 hour infusion of AmB-D and Walsh (29.40%) with L-AmB.^3^

These results represent a marked reduction in comparison with classic AmB-D therapy (Table 2). Hypokalemia seemed to be higher in our study, maybe due to the higher rate of concomitant use of other nephrotoxic drugs. Therefore, we strongly recommend potassium and magnesium supplementation, even after completing the therapy of continuous infusion of AmB-D. Hepatotoxicity was also a concern. Interestingly, one of the two patients who had the most marked increase in the liver function tests had also had a course of fluconazole treatment lasting 59 days, prior to the continuous infusion of AmB-D.

The continuous infusion of AmB-D used in our study was effective. In fact, in this small observational study, there were no deaths during the first seven days after starting the continuous infusion of AmB-D, there was no discontinuation due to lack of efficacy or extreme toxicity, and no other concomitant antifungal drugs were necessary. Table 3 presents a comparison between our data and results in the literature for AmB-D and L-AmB.

Although this was a retrospective study with a small number of patients, the results are interesting and very encouraging for us to start planning a prospective trial of continuous infusion of AmB-D in neutropenic patients with dose escalation allied to pharmacoeconomic considerations.

## CONCLUSION

Continuous infusion of AmB-D in our small retrospective experience was safe, cheap and effective. Further improvement in the outcomes from treating patients using continuous infusion of AmB-D may be possible with dose escalation. Prospective studies are needed in order to further explore the favorable pharmacoeconomic profile of this drug, and to reproduce our preliminary experience with this drug.
